# Prevention Adaptation of an Evidence-Based Treatment for Parents Involved With Child Welfare Who Use Substances

**DOI:** 10.3389/fpsyg.2021.689432

**Published:** 2021-11-19

**Authors:** Gracelyn Cruden, Shelley Crawford, Lisa Saldana

**Affiliations:** Oregon Social Learning Center, Eugene, OR, United States

**Keywords:** child welfare, parenting (MeSH), systems approach, substance use and misuse, opioid misuse, implementation science (MeSH), causal loop diagram, methamphetamine use

## Abstract

**Background:** Parental substance use, especially opioid misuse and/or methamphetamine use, is a key driver for recent increases in family involvement with child welfare and foster care placements in the United States. There is an urgent need for programs that prevent parental substance use disorders, yet few prevention programs exist that target parents’ unique needs and strengths. Adapting evidence-based treatment approaches for prevention might be an efficient, effective way to address this gap. The current study informed the rigorous adaptation of an evidence-based treatment that supports families involved with child welfare due to substance use, Families Actively Improving Relationships (FAIR), to a prevention-oriented intervention: “PRE-FAIR.” FAIR entails four treatment domains: substance use, parenting, mental health, and ancillary services (e.g., housing, medical care, and food). FAIR significantly improved parenting and reduced parental substance use in three rigorous treatment trials, but FAIR’s effectiveness in preventing the initiation or escalation of opioid misuse and/or methamphetamine use is untested. To inform adaptation, particular attention was paid to operationalizing strategies underlying a key hypothesized mediator of successful parent outcomes—engagement.

**Methods:** Graduated FAIR parents (*n* = 9) and FAIR administrators, clinical supervisors, and clinicians (*n* = 11) participated in semi-structured interviews. Content analysis was used to identify key variables driving FAIR engagement and parent outcomes. Causal loop diagramming, a qualitative systems science method, was employed to operationalize emergent themes, and describe how causal links between key variables interrelated dynamically over time.

**Results:** Themes reinforced the value of FAIR’s treatment domains for supporting parent’s sobriety and parenting skills within a prevention orientation. Ancillary supports and strong relationships were particularly crucial for helping parents cope with stressors leading to substance use. Five engagement strategies were identified as essential to parent success: 24/7 clinician availability, in-person clinician advocacy, in-home delivery, strengths-based interactions, and urinalysis. Implications for PRE-FAIR engagement strategies and dosage were identified.

**Discussion:** Traditional qualitative analyses and qualitative analyses based in systems science can inform rigorous adaptations of evidence-based treatment programs for prevention. Future research will explore additional required, fidelity-consistent prevention adaptations to FAIR, and the impact of PRE-FAIR on parental substance use and child welfare case outcomes.

## Introduction

Approximately, 7.9 million children were referred to child welfare in 2019 (U.S. DHHS and ACYF, 2020). Child foster care placements had been steadily declining for over a decade until rates began to rise in 2012, increasing over 10% through 2016 (U.S. DHHS and ACYF, 2020). Parental substance use was attributed as a leading cause of increased placements (Ghertner et al., 2018). Experiencing child maltreatment or unstable child welfare placements due to parental substance use can have both immediate and lifelong impacts on children’s mental health, physical health, and economic and social well-being (Ford et al., 2011; Jonson-Reid et al., 2019; Vanderminden et al., 2019; Strathearn et al., 2020).

Parental substance use is a prevalent, pressing public health concern. Based on the most recent national survey of non-institutionalized US adults, over 1.5 million are estimated to have experienced opioid use disorder (OUD) in the past year and over one million are estimated to have experienced methamphetamine use disorder (MUD) (SAMHSA, 2020). The co-occurrence of OUD and MUD is rising (Volkow, 2020).

Recognizing the urgent need for interventions to prevent and treat OUD and/or MUD across diverse populations, the National Institutes of Health launched the Helping to End Addiction Long-Term (HEAL) Initiative in 2017 (U.S. NIH, 2021). The HEAL Prevention Initiative, launched in 2018, aims to prevent opioid initiation or escalation of misuse among older adolescents and young adults aged 16–30 (U.S. NIH, 2021). Individuals in this age range experience the highest risk for opioid initiation, misuse, disorder, and death from overdose (Lloyd, 2018). The highest rates of opioid-related overdose fatalities in 2016 were among young adults aged 25–35, (Lloyd, 2018); individuals in this age range often are parenting and fall within the age demographic most likely to perpetrate child maltreatment (U.S. DHHS and ACYF, 2020). Indeed, recent federal statistics and a systematic literature review showed significantly increased odds of child maltreatment and child welfare involvement when parents use substances (Neger and Prinz, 2015; U.S. DHHS and ACYF, 2020). Parents at risk of OUD and MUD might best benefit from evidence-based programs (EBPs) that support their role as parents. Yet, among 52 EBPs recently reviewed for potential federal reimbursement to prevent child maltreatment among families facing high risk of maltreatment and child removal from the home, only four were found to have substantial effectiveness or likelihood of effectiveness on parental substance use (Abt Associates, 2020).

Given the limited number of EBPs for preventing substance use among parents at risk for involvement with child welfare, adapting an existing child welfare focused EBP that integrates substance use treatment is a promising, efficient, and effective approach to developing a base of prevention-oriented EBPs. One such promising program is the Families Actively Improving Relationships (FAIR) program. FAIR was developed to fill a need identified by child welfare services key informants over a decade ago. Their greatest challenges related to parental substance abuse and child neglect, and the lack of associated services accessible for parents with this profile (Saldana, 2015). Thus, an intensive outpatient treatment program was rigorously designed to address the interplay among parental: (1) substance use, (2) parenting skills, (3) mental health, and (4) ancillary needs (e.g., housing, employment, nutritious food, and medical care) (Saldana, 2015). FAIR is designed to treat parental OUD or MUD, and is delivered through an outpatient clinic supported by Medicaid (i.e., fee-for-service) (Cruden et al., 2021). Building on a decade of rigorous development, evaluation, and implementation, FAIR consistently has yielded positive and sustained effects for referred parents tracked up to 24-months (ACF 90CA1816-01-00; Saldana et al., 2013; Saldana, 2015; Cruden et al., 2021; Saldana et al., 2021). In a recently completed effectiveness trial, parents receiving FAIR showed clinical and statistical reductions in opioid and/or methamphetamine use, mental health symptoms, and parenting deficits (Saldana et al., 2021). Per FAIR clinic records from January through July 2020, over 70% of enrolled parents successfully graduated; by comparison, a recent systematic review found that only 20% of mothers involved with child welfare attend 50% or more of treatment sessions (Neger and Prinz, 2015). Although developed and tested as an intensive outpatient-treatment program, FAIR holds promise as a preventive solution for parents who are at high risk for opioid and/or methamphetamine initiation or escalation to disorder, thereby reducing risk of children’s exposure to parental substance use and neglect.

Phase one focused on building from the positive treatment effects found with FAIR to guide its adaptation to a prevention-oriented approach (PRE-FAIR). The premise was to identify ways to achieve the same high level of engagement FAIR; PRE-FAIR parents might not present with the same level of high need during treatment initiation, and therefore a different opportunity for relationship building. PRE-FAIR is hypothesized to operate more similarly to the second half of FAIR treatment, when parents are functioning with greater stability and have fostered protective factors such as strong relationships, yet still experience risk factors for substance misuse. PRE-FAIR can be conceptualized as a selective preventive intervention (Mrazek and Haggerty, 1994; Jones and Kaltenbach, 2013; Maguire-Jack et al., 2018), where PRE-FAIR parents are anticipated to respond to similar level of intervention intensity as parents experienced in the second half of FAIR treatment. Thus, the current analysis sought to understand, from the perspective of FAIR parents and clinicians, what program characteristics influenced engagement and increased stability for parents to prevent substance use during a less intensive phase of intervention. This analysis informed the adaptation to PRE-FAIR.

The goal throughout adapting FAIR to PRE-FAIR was to maintain fidelity to FAIR and anticipate strategies that could increase parents’ acceptability of PRE-FAIR (Proctor et al., 2013; Castro and Yasui, 2017). Parental engagement was selected as the initial focus of adaptation efforts because FAIR’s unique engagement strategies are hypothesized to be the key pathways through which parents agree to participate in and are retained in treatment until graduation, and through which they actively participate in setting and meeting their treatment goals (Saldana, 2015). Engagement is not just a set of activities to initiate treatment, but an ongoing set of strategies for maintaining treatment engagement and supporting graduation. Engagement can include discrete, short gestures such as encouraging text messages, as well as tangible treatment incentives such as offering a favorite beverage during treatment sessions. The level and type of engagement changes over the course of treatment, with clinicians meeting with parents daily for the first 3 weeks of treatment, and then titrating to weekly meetings over the course of 9 months. Thus, a primary objective of the current study was to characterize FAIR engagement strategies to explore whether they might need to be adapted for parents not involved with child welfare and for whom substance use is not a key driver for seeking clinical support.

A secondary objective of the current study was to explore whether and how FAIR’s four major treatment domains (substance use, parenting, mental health, and ancillary needs) would need to be modified in terms of emphasis or sequencing of delivery for PRE-FAIR, while maintaining attention to the mechanisms of core treatment components (Rotheram-Borus and Duan, 2003; Castro et al., 2010). This objective was pursued by identifying which strategies helped graduated FAIR parents maintain engagement when substance use frequency was reduced to levels similar to those expected among PRE-FAIR parents.

Two methods were used to address these objectives: traditional qualitative methods (i.e., coding semi-structured interviews), and a qualitative approach based in systems science known as causal loop diagramming.

Qualitative methods have been proposed as integral to EBP adaptation efforts (Castro et al., 2004; Castro and Yasui, 2017; Duggleby et al., 2020). They are ideally suited to validate the conceptual framework of the EBP, understand the experiences of EBP recipients and those who deliver EBPs in order to identify adaptations likely to be acceptable to these users, and to classify adaptations (Escoffery et al., 2019; Duggleby et al., 2020).

Similar to thematic qualitative analysis, causal loop diagramming identifies key variables and causal pathways that characterize behaviors. Causal loop diagrams (CLDs) shape understanding of how variables interact to produce an outcome or behavior over time through visual representation of variable interconnections and accompanying narratives (Sterman, 2000; Meadows, 2008). CLDs visually demonstrate how changes in one variable can cause changes in a second variable, and how changes in the second variable might or might not provide “feedback” into the behavior or value of the first variable (Sterman, 2000; Meadows, 2008). Feedback processes either can be reinforcing or balancing. Reinforcing feedback processes that “loop” around to continuously facilitate positive outcomes or behaviors are known as “virtuous feedback loops” or “virtuous cycles” (Sterman, 2000). In contrast, reinforcing feedback processes that perpetuate or exacerbate negative outcomes or behaviors are known as “vicious feedback loops” or “vicious cycles.” Reinforcing loops thus “enhance whatever direction of change is imposed on it” (Meadows, 2008). Balancing loops serve as checks on reinforcing loops and stabilize a system (Sterman, 2000). Understanding the key feedback processes that lead to successful intervention delivery and sustainment is a foundational step during successful EBP adaptation, with CLDs allowing for an assessment of strategies that both facilitate and hinder success (Baumann et al., 2017; Castro and Yasui, 2017; Stirman et al., 2019). Understanding both what “to do” and what “not to do” can provide a path more likely to lead to success. After laying this foundation, practitioners are better able to identify which EBP components and pathways can and should be prioritized for adaptation while maintaining fidelity to the EBP (Lich et al., 2012; Stirman et al., 2019). Further, because CLDs articulate *why* change is perceived to occur, they can be particularly helpful for specifying hypothesized mediators or mechanisms of change for an EBP, such as engagement.

Systems science methods, including the quantitative counterpart to CLDs, system dynamics simulation models, have been used as an implementation planning strategy (Leeman et al., 2017) to generate consensus among frontline workers on the policies and processes that might facilitate successful implementation of mental health and substance use EBPs (Zimmerman et al., 2016), and to address gaps in health services continuity (Huz et al., 1997). The potential of systems science methods to support other aspects of implementation planning (e.g., EBP adaptation) has yet to be fully realized. To address this gap, the current study exemplified how a systems science approach can operationalize the implementation strategy to “promote adaptability,” which focuses on identifying how an EBP can be modified to meet local needs (Powell et al., 2015).

A systems science approach was deemed appropriate to guide prevention adaptations for two reasons. First, systems science is well-suited to articulate the inherent complexity of factors elevating risk for substance use behaviors (Lich et al., 2012), similar to the complexity detailed in the FAIR logic model (Saldana et al., 2021). Second, these methods can identify dynamic feedback processes that lead to self-perpetuating positive behaviors and outcomes, such as sobriety, that should be maintained in a prevention adaptation (Galea and Vlahov, 2002; Lich et al., 2012; Mabry et al., 2013; Dasgupta et al., 2018).

In Phase 2 of this HEAL Prevention Initiative project, PRE-FAIR will be rigorously compared to standard care across parent and child outcomes using a Hybrid I effectiveness-implementation evaluation design (Curran et al., 2012). Families eligible for PRE-FAIR will be those involved in public family serving systems, including child welfare and Self-Sufficiency, with parents who are at risk for but do not have current OUD or MUD diagnoses, yet experience current risk factors for OUD and MUD similar to those experienced by the FAIR sample, including unmet ancillary needs, a history of trauma, exposure to individuals who misuse substances, and untreated mental health disorders (Saldana, 2015; Saldana et al., 2021).

## Materials and Methods

### Procedures

Key informant interviews elicited the perspectives of graduated FAIR parents and FAIR clinicians and administrators (Israel et al., 2005). Queries emphasized gaining an understanding of the strategies used to facilitate and maintain clinical engagement, and which strategies might be improved or modified to serve a prevention-focused population. Interviews were qualitatively analyzed to inform the design of CLDs describing how FAIR treatment components and engagement strategies led to positive parent outcomes. CLDs thus informed how treatment components and engagement strategies might be maintained or modified to support effective FAIR prevention adaptations.

### Semi-Structured Key Informant Interviews

Semi-structured interviews with graduated FAIR parents (*n* = 9) and current or previous FAIR clinicians and administrators (*n* = 11) were conducted. Graduated FAIR parents were included because they experienced treatment during the phase of session frequency similar to the expected level of contact for PRE-FAIR parents. A multi-stage process was used to identify eligible parents in order to protect parent confidentiality and well-being. First, FAIR clinicians were asked to recommend parents who had graduated at least 1 year prior. Referred parents were contacted by their FAIR clinician to obtain permission for the study’s Principal Investigator and FAIR developer (Saldana) to contact them. The clinicians were not involved further or told if their referral participated. The Principal Investigator contacted parents directly, explained the study purpose, and introduced the parent to the interviewer (Cruden). Upon agreeing to participate, parents were mailed consent documents and instructions for joining the interview on a video conferencing platform. Interviews lasted approximately one-hour each. Parents were compensated $50 for their time via their choice of a personal check or gift card to a local store, where they could obtain daily necessities (e.g., food and gas). Throughout recruitment, consent, and the interviews, parents were reminded that the interviewer was not involved with the FAIR clinic or team and that their responses would be kept confidential.

All current FAIR clinicians (*n* = 7) and administrators (*n* = 2) were invited and agreed to participate. Previous clinicians who worked with FAIR recently were invited and consented (*n* = 2). Clinicians were compensated $50 via a personal check. Similar to parents, clinicians were given instructions for joining the video conferencing platform and consent documents prior to their interview.

Interview scripts were co-created by the interviewer and FAIR developer. Graduated parent interviews focused on the parents’ perception of the services they received through FAIR, services that parents accessed with the support of their FAIR clinician, current strategies for maintaining sobriety, and suggestions for adapting FAIR to PRE-FAIR or generally improving FAIR. Clinician and administrator interviews focused on the ancillary services that they helped parents to access, barriers in connecting parents to services, suggestions for adapting FAIR to PRE-FAIR, and engagement strategies. Interviews were recorded and professionally transcribed for qualitative analysis in Dedoose™ (Dedoose, 2016). Study procedures were reviewed and approved by the Oregon Social Learning Center Institutional Review Board.

### Qualitative Analysis

Content analysis was employed to derive key variables for the CLDs (Mayring, 2015; Marçal et al., 2021). A hybrid approach with both inductive (data-driven) and theory-driven coding was applied across a multi-stage process (Fereday and Muir-Cochrane, 2006). First, a codebook was drafted based on the FAIR logic model (Saldana et al., 2021) and study goals (e.g., prevention adaptation implication) by the interviewer and Principal Investigator. Next, the interviewer added initial codes based on interview memos (e.g., parent feels seen, dosage, and value of FAIR). The interviewer then trained two independent coders who were naïve to detailed participant characteristics beyond the fact that participants had graduated from FAIR or work as a clinician/administrator for FAIR. The independent coders were aware that the study purpose was to derive feedback loops and adapt FAIR for prevention, as these study goals were deemed important to focus the coding and derive rich information about feedback behaviors. To test the reliability of the codebook, coders applied the initial codes and identified additional emergent codes across representative transcripts (*n* = 4) (Creswell and Zhang, 2009; Crowe et al., 2015). The three coders met to discuss discrepancies and converge emergent codes. The codebook then was refined and independently applied by all three coders to all transcripts. Codes were not mutually exclusive. Each coder kept detailed coding memos and reviewed other coders’ memos before peer-debriefing; coders met two additional times for peer-debriefing to reach consensus (Patton, 2001; Levitt et al., 2018). Codes then were compared and contrasted to cluster them into meaningful groups in a computer spreadsheet (Patton, 2002; Ritchie and Spencer, 2002). The frequency with which codes had been applied and coding memos guided identification of salient themes. A theme for each group was created to characterize the content represented by code clusters and reviewed during a final peer-debriefing (Glaser, 1965; Patton, 2001). Themes were operationalized through CLD narratives (section “Deriving Causal Loop Diagrams”).

Coding consistency was validated by comparing the overall frequency of applied codes using the coding matrix available within Dedoose™. Coders applied proportionally equivalent codes across transcripts. To support the dependability of results (Crowe et al., 2015), the FAIR manual and developer were consulted to validate the codes and associated definitions for each code.

### Deriving Causal Loop Diagrams

Causal loop diagrams were created to visually depict how salient themes interrelated, with a focus on how key FAIR treatment and engagement strategies related to parent outcomes. A table was created to identify the variables within each loop (with variables often drawn from a qualitative code), tell a story describing the loop based on qualitative themes and causal links identified in coding memos, and include representative quotes for each story (Hovmand, 2014; Baugh Littlejohns et al., 2018; Marçal et al., 2021). Particular attention was given to explaining the pathways (i.e., intervention strategies) through which engagement in FAIR was achieved and maintained in order to identify those to replicate or modify in PRE-FAIR. Engagement was broadly conceptualized as parent attendance in treatment sessions and service appointments. The two trained coders reviewed the table and CLDs for accuracy and consistency with qualitative data. CLDs were designed in Stella Architect v 1.9.4 (ISEE Systems, 2019).

## Results

### Sample Characteristics

One dad and eight moms were interviewed. Due to the sensitive nature of the interview topic and small sample size, additional parent demographic data was not collected. To protect clinicians’ and administrators’ confidentiality, limited demographics are presented. The sample consisted of nine clinicians and two administrators (who did not interact directly with parents). One current clinical supervisor and one previous clinical supervisor participated. The majority of clinicians were licensed as Qualified Mental Health Associates (QMHA) and the minority were licensed as Qualified Mental Health Professionals (QMHPs). QMHPs hold a minimum of a Master’s degree and experience. Clinicians’ experience with FAIR ranged from less than 1 year to approximately 10 years.

### Operationalizing the Engagement Strategies in Families Actively Improving Relationships and Strategy Links to Parents’ Sobriety

**FIGURE 1:**
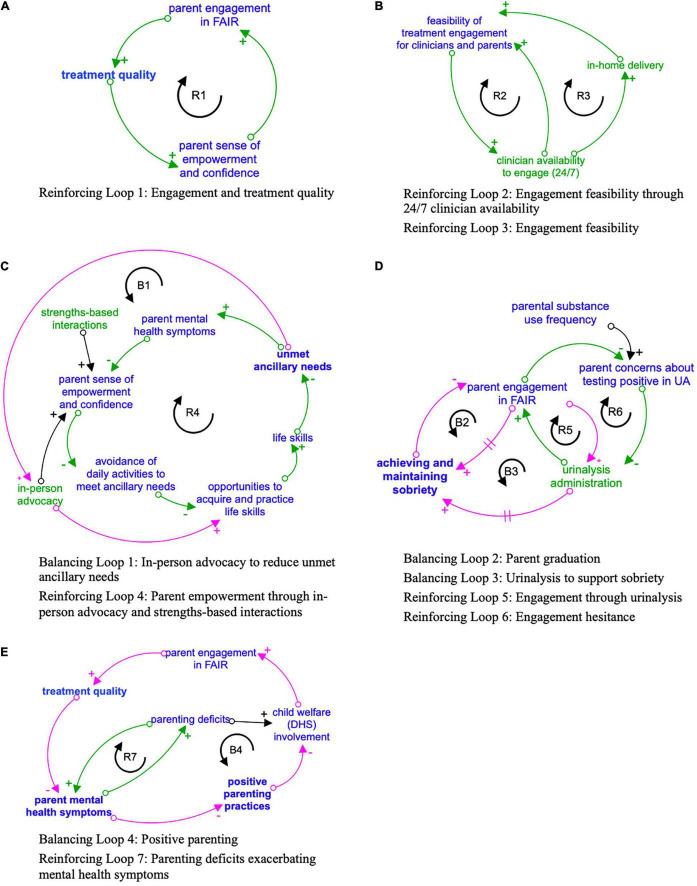
**(A-E)** Key feedback loops driving the causal theory of how FAIR engagement strategies impact parent engagement and treatment goals. Engagement strategies designated in green. Outcomes related to the four primary FAIR treatment domains designated in bold. Reinforcing loops indicated in green with R clockwise arrow, and Balancing loops in pink with B counter-clockwise arrow. Arrows with a + sign indicate that the variables either both increase or both decrease when there is a change. Arrows with a – sign indicate that as one variable increases, the other decreases, or vice versa. A hashmark (two parallel lines) on an arrow represents a delay in the effect of the first variable on second variable.

**FIGURE 2:**
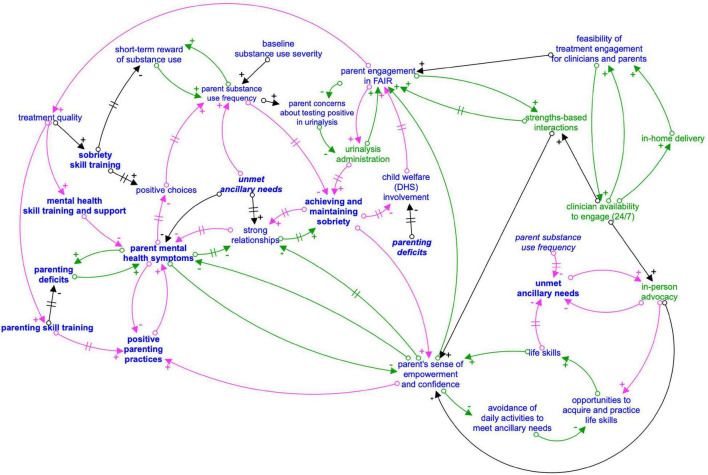
Composite causal loop diagram: FAIR engagement strategies and impact on parent treatment goals. Arrows with a + sign indicate that the variables either both increase or both decrease when there is a change. Arrows with a – sign indicate that as one variable increases, the other decreases, or vice versa. A hashmark (two parallel lines) on an arrow represents a delay in the effect of the first variable on second variable. Foodback loops are indicated with like colored arrows (green for reinforcing and pink for balancing). Variables in green represent engagement strategies. Outcomes related to the four primary FAIR treatment domains designated in bold. Italicized variables are repeated from another part of the causal loop diagram for visual simplicity.

Key feedback loops driving the causal theory of how FAIR engagement leads to parents achieving and/or maintaining sobriety are presented individually (Figure 1) and in a more comprehensive CLD (Figure 2) showing how feedback loops interrelate. A loop describing how engagement drives treatment quality is first presented. Next, detailed loops that explain the dynamics driving engagement are presented. For each loop, the qualitative themes that characterized each loop are first presented, followed by descriptions of how loops relate to parent treatment goals.

The comprehensive CLD (Figure 2) shows feedback loops that are important for appropriately characterizing how FAIR and PRE-FAIR dynamically operate to help parents make positive choices about their health and parenting practices. Given the study purpose of informing a prevention adaptation, this CLD does not entail the full extent of dynamic complexity related to the original FAIR treatment program, designed for a high-needs population. The Supplementary Material contains examples of these more complex feedback loops, including loops that demonstrate the endogeneity or interconnectedness of FAIR’s four treatment domains (substance use, parenting skills, mental health, and ancillary needs).

#### Central Reinforcing Feedback Loop: Engagement and Treatment Quality (Figure 1A)

Engagement emerged as a key virtuous feedback loop driving treatment quality. Emergent themes suggested that clinicians engaged parents through five key strategies: 24/7 clinician availability, in-home delivery, in-person advocacy, strengths-based dialogue or interaction, and urinary analysis (UA). Each strategy is detailed below [section “Engagement Strategy Feedback Loops: 24/7 Clinician Availability (Figure 1B)” to section “Engagement Strategy Feedback Loops: Urinalysis Administration (Figure 1D)”]. As parents received high-quality, consistent engagement strategies from clinicians, they were more likely to increase participation in FAIR (i.e., engagement) through honest communication and regular attendance, leading to high-quality treatment (Figure 1A). High-quality treatment increased parents’ sense of empowerment and confidence due to strengths-based treatment delivery [section “Engagement Strategy Feedback Loops: Strengths-Based Interactions (Figures 1A,C)”] of evidence-based, manualized treatment components such as parenting skills training and positive coping strategies. As parents felt empowered and progressed toward their treatment goals, they increased their desire to engage with their clinician, and this engagement helped parents continue making positive choices about their health and sobriety. Demonstrating the strength of the therapeutic relationships and engagement quality, several parents reported wanting to continue engaging their clinicians after graduating FAIR. The following quote illustrates this: “I reach out to her because I really, I enjoyed the program. I’ve been through multiple A&D (alcohol and drug) programs, and so this was—I like the bonding.”

#### Engagement Strategy Feedback Loops: 24/7 Clinician Availability (Figure 1B)

##### Components and causal pathway(s) to engagement

Parents could reach their clinician or another FAIR clinician at any time of day on any given day (i.e., 24/7). This consistent availability was made feasible through team-based clinical coverage, with a designated clinician on-call should the parent’s primary clinician be unavailable (e.g., vacation and sick leave), and weekly team meetings. These meetings helped ensure that all clinicians were aware of pertinent details related to other clinicians’ parent treatment plans. Clinicians used clinic-provided phones to frequently and directly communicate with parents. These strategies increased the feasibility for clinicians to consistently engage with parents (Figure 1B). As part of 24/7 engagement, FAIR clinicians reached out to parents between formal treatment sessions to let parents know they were thinking about them, provide strengths-based support [see section “Engagement Strategy Feedback Loops: Strengths-Based Interactions (Figures 1A,C)”], and to reinforce that parents can reach out at any time if they need support, including if they were considering using substances. Both clinicians and parents reported that this consistent engagement helped build rapport. One parent gave an especially clear example of how 24/7 availability helped them engage in FAIR and make positive choices about their sobriety and children’s well-being (of note, parents and clinicians both normally refer to child welfare services as “DHS” because the child welfare department is housed within the Department of Human Services):

I mean I even called [counselor] at 11:00 one night and just told her that I was 99% sure my boyfriend was high and that they had said they’d come if it was an emergency. They were 24/7. She said, “I think maybe we can wait until morning. It’s 11:00.” I said, “No, it’s an emergency, and if DHS were to show up for any reason right now, they would take the kids.” [Another FAIR clinician], was my boyfriend’s counselor, so he came over at 11:30 at night and he [unintelligible], and took him to a motel to get him out of here because he was dirty. Just no other treatment would do that.

##### Link(s) to treatment goals

As parent-clinician rapport grew, parents increased their belief that clinicians would help them meet their treatment goals. Increased rapport thus increased parents’ willingness to consistently and honestly engage. Increased parent engagement improved treatment quality by providing more opportunities for the parent to acquire sobriety skills, which in turn increased the likelihood that parents consistently made positive choices about their substance use. As one parent reported: “I mean any time of day if I needed to text or call, I could call or text. It wasn’t a 2-day waiting period. They’d get back to me instantly even if it came down to, ‘Well, I just had a dream about using and now I want to use.”’

#### Engagement Strategy Feedback Loops: In-Home Clinician Availability (Figure 1B)

##### Components and causal pathway to engagement

Because clinicians were available 24/7, they were better able to accommodate parents’ schedules and meet them where they live, even if unhoused, and otherwise spend their time. In-home engagement increased the feasibility of clinician engagement and parent engagement, thereby increasing opportunities to engage in FAIR, receive high-quality treatment, and achieve treatment goals (Figure 1B). Straightforwardly, it was more feasible for a parent to engage because they do not have to obtain independent transportation or commute. Parents appreciated this accessibility: “It just made it easier. I think I was first starting to become a manager in the middle of us meeting so I was starting to work a lot of hours, and a lot of long hours so that was really hard on me to begin with so [counselor] would just meet me here which was really nice too. It’s easy. A lot of the times, I would be feeding the baby breakfast when he came or whatever.” Further, having clinicians in their home sometimes helped parents to honestly engage: “I just think I felt more comfortable in my home anyway… I mean, that was really nice for him to see me in my own personal setting I think. It’s harder to lie and hide in those kinds of settings.”

##### Link(s) to treatment goals

In-home engagement directly facilitated parents’ treatment goals through at least two pathways. First, in-home engagement provided a comfortable environment for parents to honestly engage and to collaboratively identify individualized treatment goals and potential challenges to reaching those goals with their clinicians.

A big part of it was – a lot of treatment facilities, you get thrown into a group setting with a whole bunch of people. So, the thing that helped a lot was the flexibility of being able to meet in different locations. If you can’t make it, then they would come to you. Sometimes we’d meet at parks or we go out to lunch for our meetings. Different things like that to make me feel comfortable, and that made it a lot easier to open up.

Second, in-home engagement offered the opportunity for clinicians to deliver highly personalized, and at times non-traditional, treatment or engagement strategies that increased parents’ desire to engage with FAIR. A small, creative gesture through in-home delivery can provide long-lasting support and eventually increase engagement through encouraging the parent to engage rather than choosing to discontinue treatment, as demonstrated by the following reflection:

Most of the time, we met at my house, but she’d meet me wherever I was at. There was a few times that I was trying to get out of meeting her because I didn’t want to take the UA, and she was just telling me, “It doesn’t matter where you are, I’ll meet you there. I’ll drive to you. Just tell me the spot.” It was one of those couple of times that I didn’t show up to the house, and she left me a note, and I actually still have the note. It just said, “I know you’re having a hard time. You can do this. Hang in there. Please call me.” She even made me a Superhuman Mom Strength Award that she cut out and made herself, and I still have it on my board on my wall.

From the clinician’s perspective, in-home engagement provided opportunities to practice skills discussed during treatment sessions (such as positive parenting skills), directly observe parents’ environment and interpersonal interactions (such as with their children or partners), and identify treatment strategies that might not otherwise have been identified. A clinician reflected:

Whereas, one of the advantages of what we have is we meet parents literally where they’re at, and going into their home turf and seeing what it is that they have to deal with. That can provide a lot of advantages when we get eyes on the situation. Maybe we notice something that they maybe don’t notice because they’ve just become accustomed to it. That just helps everything run a little bit better. Whether it’s like “Hey, what if we rearrange your furniture so that it felt like this was a different room instead of being stuck in the room where you used to use?” …It doesn’t cost anything to do that. Just time.

#### Engagement Strategy Feedback Loop: In-Person Advocacy (Figure 1C)

##### Components and causal pathway(s) to engagement

When providing in-person advocacy, clinicians assisted parents with completing a range of daily life activities, such as navigating ancillary services (e.g., medical care, long-term mental health treatment, housing, employment, and child care) and completing DHS case management and legal sessions (e.g., court). Similar to in-home engagement, in-person advocacy was a standard FAIR treatment component that was tailored to parents in a key balancing feedback loop (Figure 1C) that helped disrupt vicious feedback loops that might lead to parental substance use, such as increased mental health symptoms. For example, parents reported sometimes experiencing negative interactions with ancillary service providers, such as physicians. These experiences caused parents to have anxiety when accessing these services and to believe that they will not be successful obtaining services. As a result, parents were less likely to access services. When parents faced challenges accessing services, the resulting unmet needs increased parents’ stress and anxiety, leading to a decline in their mental and physical health and sobriety. This vicious cycle of parental mental health symptoms increasing due to unmet ancillary needs could be disrupted through in-person advocacy by the clinician. One parent reflected on how in-person advocacy disrupted this vicious cycle:

I got an amazing counselor. She was able to help me through pretty much everything. Because at the time, I still had a DHS case going, and she was able to help me with the problems I was having as far as – she was able to help me with everything from taxes to finding other treatment facilities that could help me for when I was finished working with FAIR. Pretty much anything that I had wrong in my life, she went out of her way to make sure that I had some sort of resource to help me get through it so I wasn’t just up in the air, stressing about anything.

Further, in-person advocacy provided opportunities for clinicians to support parents as they worked toward meeting DHS (i.e., child welfare) requirements. Parents often discussed relying upon clinicians to “interpret” DHS requirements and advocate for them to ensure that DHS understood the progress parents were making. These parents reported that DHS would at times adjust expectations and DHS treatment goals accordingly. This advocacy helped parents “feel seen” and supported by the clinician, and thus more willing to engage with their FAIR clinician and DHS caseworker. The impact of in-person advocacy can be seen in the following parent quote:

For one, the biggest help was like another voice. Someone who could communicate between the two, be it a worker, me, and the kid’s attorney. Just to help get information to people that maybe the case worker wouldn’t say it in the correct way because I wanted it translated. And then, being someone with that firsthand knowledge of what was going on, being able to communicate to those people.

By advocating for parents directly to those who provide ancillary services, clinicians helped parents feel supported and directly increased parents’ access to ancillary services through changing the interpersonal dynamics between parents and ancillary service providers (Figure 2):

I’ve seen it where I’ll go with parents, just having another professional there, having an advocate who can say that “No, this person is trying to go legit. This person is working a program. There’s an accountability piece here. This is not like the other folks that you may have seen.” Sometimes, that can be a huge game changer. I’ve seen it even to the point where someone like a doctor who starts the first appointment and they got kind of an attitude toward the parent. Having not even met them before, just because of whatever preconceived notions they have about what they’re coming in for and what to expect with that. By the third appointment, they’ve got a completely different attitude and they’re asking them about what all is going in with life and now they’re treating them like a human.

Parents’ experiences directly mirrored this clinician’s perspective:

It was helpful. [counselor] was my FAIR counselor, but he went with me to a lot of the doctor’s appointments which I’m glad because the doctor – I mean I get it. He sees many people, who are in there, just trying to get things to get high on. I was there because I was trying to do it the right way, I guess. At first, it was rough. He was kind of mean, but – [counselor] would talk about it and things got smoother from there. The doctor knew I was serious. It just got better from the first appointment on.

##### Link(s) to treatment goals

In-person advocacy created a balancing loop by increasing opportunities for clients to access ancillary services and practice life skills, such as advocating for themselves, eventually reducing the need for in-person advocacy as ancillary needs were met. This balancing loop strengthened the virtuous reinforcing loop in which parent empowerment led to reductions in parent mental health symptoms and unmet ancillary needs, thereby further empowering parents (Figure 1C). One clinician described this link as follows:

So, if they’ve got a DHS case and the DHS case workers are able to provide some of those resources but maybe the parent doesn’t know how to ask for that or has had some trouble with the relationship between them and the case worker, so then accessing that feels awkward… But we also try in the same sort of area of their DHS cases or things along those lines, if it’s meeting with the parole officer, we’ll try and support them in that. Maybe go to one or two of those meetings just to let them know they’re okay, they’re doing a program and this is the program, and of course, we get our allies and everything up and running before we actually make that meeting happen. But sometimes that can be a big game changer as far as not only making the access of that resource go better, but then moving forward, what that relationship looks like can be quite different.

From the parent’s perspective, in-person advocacy was essential to feeling supported in the moment (empowered), but also for increasing opportunities for learning and applying life skills that can serve them over the long-term, such as emotion regulation and positive coping techniques. These skills then served parents in both the short-term (e.g., DHS case) and long-term (e.g., ongoing positive interpersonal relationships and accessing ancillary services). The following quote demonstrates how essential in-person advocacy was for one parent to practice some life skills:

Sometimes, they DHS would say stuff that you don’t understand or just to have that one support person saying that you are doing what you’re supposed to be doing and you have that one person in your background. That helped a lot because then, they weren’t just listening to what I was saying. They had someone else backing me up that I was actually doing what I was supposed to be doing… I’m one of those people that if I feel like you’re attacking me, I get defensive very much. It’s a fight or flight thing. I either fight back. That’s my thing. I just fight back. I get angry or I get upset. I cry. I shut down. Having [clinician] there, he was there to bring me back like down and ground me and show me grounding tricks and how to do it in the moment instead of just telling you how to do it.

Relatedly, graduated FAIR parents reported that meeting their ancillary needs was integral to preventing substance misuse, as demonstrated in the following quote:

A lot of substance abuse problems come from stress and people trying to deal with stress in their own way, and a lot of that comes from people who just need help. Whether they’re going through financial troubles or anything like that, it’s like a lot of the things that they helped me with, at the core, solves my addiction problem too. It can help a lot of people that aren’t even going through addiction but just need help learning how to cope with different problems in their life.

#### Engagement Strategy Feedback Loops: Strengths-Based Interactions (Figures 1A,C)

##### Components and causal pathway(s) to engagement

Strengths-based interactions emphasized communicating parent’s positive choices and behaviors. Similar to in-home delivery and in-person advocacy, strengths-based interactions are a standard FAIR treatment strategy. The use of strength-based interactions increases with increased parental engagement because there are more opportunities for such interactions. As parents felt supported and not judged by the clinician, they were more likely to share their experiences honestly and increasingly engage with FAIR. High-quality, increased parent engagement provided opportunities for the clinician to understand what types of treatment the parents were receptive to and could benefit from, parents’ treatment goals, and challenges parents faced. Clinicians could then tailor engagement and treatment strategies instead of taking a one-size-fits-all approach. For example, one parent characterized themselves as unorganized, and described how their therapist brought them a notebook to organize their paperwork as they collaboratively worked toward meeting the parent’s ancillary needs and other treatment goals. The strengths-based, tailored strategies helped parents feel supported and empowered. As one parent reflected: “It’s just a great place to help. Instead of your life being controlled and put in place by somebody else and you just following orders, they teach you how to put your life in order.”

Further, hearing positive comments about their choices and unique strengths helped parents to see value in their skills, experiences, and emotions, which also increased their sense of empowerment and confidence. Parent empowerment was so integral to positive parent treatment outcomes that it is present in multiple reinforcing and balancing feedback loops (Figures 1A,C, 2). One parent described how strengths-based engagement empowered them: “Well, he [clinician] explained it in a way where it didn’t feel like it was “I’m better than you” type of thing. It’s like “I’m teaching you these coping tricks. I’m teaching you these things so you can have a better life.” It wasn’t just “I’m teaching you these as a paycheck.” It was “I’m teaching you these so you can do better because I know you can do better.” As parents felt empowered, they saw how they could make positive choices about their health with the support of FAIR, which made them want to initiate and maintain engagement. Several clinicians explained how strengths-based engagement helped them identify treatment goals with parents in a manner that parents could positively internalize, such as the following:

I always try to do the sandwich approach when I’m talking with families and just really start it off with like praise and kudos… and then I kind of go on to the hard topic because sometimes I’m able to – depending on the rapport I have with somebody, I’ll hit him with just transparency and call on them on their behavior, but then I’ll follow it up with like more positives and praise and clients are – they receive it.

##### Link(s) to treatment goals

Empowerment was not only a key pathway to FAIR engagement (i.e., short-term positive outcome), but also a key pathway to supporting parents as they built a long-term sense of confidence and similar internal supports. Several parents honed in on this pathway when asked about the value of FAIR, such as the following:

When there are so many steps that they do with you that you get your own self-worth back. It’s to know that you are actually worth something or that you do have potential. Do you know what I mean? It’s like they build your confidence up as well. It’s not just like…they’re just there. Like I said, not a lot of people have people that are just actually there. They were just there and I needed that.

Strengths-based interactions thus facilitated a key virtuous feedback loop in which parents maintain sobriety and parent’s sense of empowerment. Notably, the importance of receiving positive (i.e., strengths-based) support was the most commonly applied code among FAIR treatment strategy implications for parent outcomes.

#### Engagement Strategy Feedback Loops: Urinalysis Administration (Figure 1D)

Urinary analysis is administered regardless of severity of substance use or a parent’s time in FAIR (Saldana, 2015). While UA contributes to both engagement and thus treatment, creating a reinforcing loop (Figure 1E), UA administration also declines as parents achieve sobriety and graduate from FAIR, introducing a balancing loop. A second reinforcing loops describes how some parents avoided engaging in FAIR initially because they were concerned about a positive UA. These concerns can be mitigated through treatment and engagement strategies, described below.

##### Components and causal pathway(s) to engagement

UA administration is a recurring opportunity to engage parents in a discrete treatment activity (i.e., monitoring substance use). It is coupled with a strengths-based interaction and evidence-based strategies such as contingency management (Saldana, 2015). Contingency management in FAIR is operationalized through “FAIR Bucks” that can be redeemed at the FAIR Store for everyday household items, clothes, toys, and other items of interest to parents and their families. Parents might have been offered FAIR Bucks for providing a UA early in treatment when they were more likely to provide a positive sample. Parents reported that the consistency of UA administration, coupled with strengths-based interactions regarding UA results, helped them to feel accepted by clinicians and realize that they could honestly engage: “I appreciate the positive reinforcement and how they didn’t degrade me if I did have a dirty UA.”

##### Link(s) to treatment goals

Some parents reported that UA administration directly supported them in achieving and maintaining sobriety by providing a consistent, unbiased, tangible source of accountability and measure of treatment progress. As one parent described: “I mean, the accountability was really nice too. Some people might be mad about or are like negative feeling I guess about being drug-tested, but I’ve enjoyed it because I liked the accountability behind it. I mean at first, you almost don’t trust yourself to stay clean and stuff, so just knowing that you’re going to have to take those, it helps you as well to get over that hump.”

#### Moderating Variable of Substance Use Feedback Loop: Baseline Substance Use

Not all parents immediately engaged with FAIR. Parents who had more severe substance use at baseline reported delayed engagement in FAIR. While not a focus of the current study, parents in this sample reported behaviors consistent with previous research indicating that substance use can provide short-term positive reinforcement for parents, which causes them to continue using (Figure 2; Han et al., 2018; Volkow et al., 2019). FAIR treatment strategies aim to interrupt this vicious feedback loop. Baseline substance use can thus moderate FAIR engagement timeliness and quality until a parent has experienced sufficient consistent clinician engagement strategies, or external stressors such as involvement with child welfare. Through engagement and high-quality treatment, parent substance use frequency declines over time in FAIR (Saldana, 2015; Saldana et al., 2021). Note, parental substance use is included in the CLD as “substance use frequency” to emphasize that substance use frequency can dynamically change over time, regardless of baseline severity, which includes frequency and dosage of use.

I think we [FAIR counselor] worked together for almost a year. The first part of it, I wasn’t ready to get clean and so I’m just trying to push him away but he would not leave me alone. [Laughter] So, then finally – I was pregnant during this time too, and so I gave birth to my son, and they took him right away and so we had court a couple of days after that and he showed back up. I told him I was ready and so he stuck it with me until I got [into detox].

Clinicians reported the importance of engagement strategies to reduce or overcome parents’ initial rejection of FAIR. For example, one clinician reflected on the importance of being consistent and persistent:

The most important thing for me in FAIR is engagement. You have to engage with those parents. I’ve had parents fire me, and then I say, “Well, I’ll see you tomorrow,” and they’ll say, “Okay.” [Laughter] They’ll get mad at me for whatever reason. Sometimes it’s not my fault. They’re mad at me because I’m the one there, and they’ll say, “I hate FAIR, I don’t want to be part of FAIR anymore,” and I’ll say, “Well, we can talk more about it tomorrow.”

### Interconnections Between Engagement and Treatment Strategies to Support Parents’ Positive Choices

Families actively improving relationships engagement strategies, while planned as part of families actively improving relationships manualized approach, can be delivered more or less intensely as parent engagement varies over time (i.e., parents must engage to receive some treatment and experience further engagement strategies). Further, interview themes pointed to the endogeneity of parent’s success—improvement in one domain, such as reduced mental health symptoms, led to improvements in other domains such as positive parenting practices. As the current study was focused on specifying potential adaptations to FAIR for PRE-FAIR, and the phenomenon of parental improvement in one treatment domain affecting another domain has been observed in previous FAIR trials as well as systematic reviews of the literature (Neger and Prinz, 2015; Saldana et al., 2021), detailed presentation of these results can be found in the Supplementary Material. Figure 2 represents how the individual feedback loops presented in section “Operationalizing the Engagement Strategies in Families Actively Improving Relationships and Strategy Links to Parents’ Sobriety” and depicted in Figure 1 are interconnected and situated within the larger system of FAIR treatment strategies and parent outcomes.

## Discussion

Table 1 offers an overview of how key engagement strategies and treatment component feedback loops relate to prevention adaptations. Results suggest that PRE-FAIR clinicians should maintain fidelity to the FAIR model of synergistically delivering all four treatment domains (substance use, parenting practices, mental health, ancillary needs; Table 1 and Supplementary Material) and using engagement strategies such as a strengths-based approach to support virtuous cycles of parent success (e.g., improvements in mental health and positive parenting, Figure 1E). However, engagement strategies directly and indirectly affected parent success across each of these domains, highlighting the need to consider how any engagement adaptations for PRE-FAIR might have cascading effects on parent engagement and treatment outcomes (section “PRE-FAIR Engagement Timeline Variation by Baseline Substance Use”). Thus, adaptation effects will be tracked carefully in the PRE-FAIR trial.

**TABLE 1:** Key feedback loops for parental engagement in FAIR: Implications for prevention adaptation to PRE-FAIR.

Key feedback loops	Causal link to core FAIR treatment domains and outcomes (substance use/sobriety, parenting, mental health, and ancillary needs), FAIR engagement, and treatment quality	Implications for prevention adaptation
**FAIR engagement** increases **treatment quality** (Figure 1A)	Direct: Treatment quality Indirect: Parent empowerment	Emphasize importance of engagement and creative, multi-pronged approaches to engagement when training new FAIR clinicians (section “Maintain and Track Creative, Multi-Strategy Engagement”)

**Engagement feasibility** increases with **24/7 clinician availability** and **in-home delivery** (Figure 1B)	Direct: FAIR engagement Indirect: Treatment quality; achieving or maintaining sobriety; parenting practices; mental health; and ancillary needs	Emphasize importance of engagement and creative, multi-pronged approaches to engagement when training new FAIR clinicians (section “Maintain and Track Creative, Multi-Strategy Engagement”)

**In-person advocacy** reduces **parent mental health symptoms** (Figure 1C)	Direct: Ancillary needs; Mental health Indirect: Achieving or maintaining sobriety; parenting practices; FAIR engagement; and strong relationships	Prioritize meeting parents’ ancillary needs (section “Prioritizing Ancillary Needs Treatment Component”)

**Strengths-based engagement** increases **parent empowerment** to reduce ***unmet ancillary needs*** (Figure 1C)	Direct: Life skills, ancillary needs Indirect: Parenting practices; mental health; and FAIR engagement	Emphasize importance of engagement and creative, multi-pronged approaches to engagement when training new FAIR clinicians (section “Maintain and Track Creative, Multi-Strategy Engagement”) Prioritize meeting parents’ ancillary needs (section “Prioritizing Ancillary Needs Treatment Component”)

Increased **baseline substance use** severity delays high-quality **parent engagement** in FAIR (Figure 1D)	Direct: FAIR engagement Indirect: Treatment quality; achieving or maintaining sobriety; parenting practices; mental health; and ancillary needs	Modify treatment dosage and titration for PRE-FAIR (section “PRE-FAIR Engagement Timeline Variation by Baseline Substance Use”)

**Child welfare involvement** increases **parent engagement with FAIR** (Figure 1E)	Direct: FAIR engagement Indirect: Treatment quality; achieving or maintaining sobriety; parenting practices; mental health; and ancillary needs	Modify treatment dosage and titration for PRE-FAIR (section “PRE-FAIR Engagement Timeline Variation by Baseline Substance Use”)

Ancillary supports (**unmet needs)** increase likelihood of **achieving or maintaining sobriety** (Figure 2)	Direct: Achieving or maintaining sobriety Indirect: Parenting practices; mental health; ancillary needs; and FAIR engagement	Prioritize meeting parents’ ancillary needs (section “Implications for Prevention Adaptations and PRE-FAIR Trial” to “Future Research”)

*Loop variables are indicated in bold.*

The current study offers three primary implications for PRE-FAIR: (1) the need to continue employing creative, multi-strategy engagement; (2) the role of baseline parental substance use on expected PRE-FAIR treatment duration and dosage; and (3) the need for prioritizing ancillary needs earlier in PRE-FAIR treatment compared to FAIR. These lessons could be generalized to guide adaptations for treatment programs similar to FAIR and testing or implementing adapted programs in new settings or locations.

### Implications for Prevention Adaptations and Prevention-Oriented Approach Trial

#### Maintain and Track Creative, Multi-Strategy Engagement

The first key implication for PRE-FAIR is the importance of parent-centered, multi-strategy engagement. FAIR engagement strategies increased the likelihood of parents successfully achieving proximal treatment goals and led to the creation of long-term supports. Clinicians reflected on how they appreciated the ability to be creative, such as bringing a parent’s favorite drink to a treatment session, in order to build rapport and increase parents’ honest engagement. Findings around clinicians’ engagement strategies are consistent with recent studies that report the need for off-business hours clinician availability and persistent engagement to develop therapeutic relationships with parents involved with child welfare (Yoon et al., 2021). Based on the current study, it is evident that creative, consistent engagement strategies also will be used for a prevention-oriented intervention, but qualitatively different creatives strategies might emerge during PRE-FAIR. Thus, PRE-FAIR trial procedures will be designed to capture this creativity and identify potential new strategies unique to PRE-FAIR. In particular, strengths-based engagement still will be essential for PRE-FAIR, as illustrated by a clinician’s reflection: “Prevention really comes from, in my mind, just an overall sense that there is somebody out there to help and that choice to reach out to those people if they even know that they exist, right? Then, feeling at least, hopefully, the confidence to be able to reach out.”

Of note, PRE-FAIR parents might be at risk for involvement with child welfare, but might not yet have an active case. Some of the interviewed graduated parents in FAIR reported that child welfare involvement was an impetus for their engagement in FAIR (Figure 2). Consistent, creative engagement by PRE-FAIR clinicians might be even more important in PRE-FAIR to build rapport and parent engagement in the absence of service system-level consequences such as removal of one or more children from the home.

#### Prevention-Oriented Approach Engagement Timeline Variation by Baseline Substance Use

The second key implication for prevention is the potential appropriateness of PRE-FAIR and therefore duration of PRE-FAIR engagement given a parents’ baseline substance use. Baseline substance use was reported to potentially moderate parents’ engagement in FAIR, as parents with higher levels of substance use often had a longer period of initial engagement in FAIR. Clinicians reported that parents with higher baseline levels of substance use often have more complex ancillary needs, requiring additional time to help parents meet those needs. Thus, parents in PRE-FAIR, who by definition will have lower levels of baseline substance use than those in FAIR, are hypothesized to have shorter overall treatment duration. This insight led to modifying the planned treatment dosage for FAIR, given that the five engagement strategies (i.e., 24/7 availability, strengths-based approach, in-person advocacy, in-home delivery, and UA) might be delivered more immediately (i.e., shorter delay from initial parent engagement to high-quality engagement) and less frequently (i.e., less frequent parental substance use, thus less need to engage and disrupt that vicious cycle) in PRE-FAIR.

The PRE-FAIR dosage schedule was modified from FAIR to be 3 days per week in the first month of parent participation instead of 5 days per week, with planned titration to 2 days per week in months two through three, and 1 day per week in month four, upon which parents are anticipated to be graduating from PRE-FAIR. This is a notable, yet fidelity-consistent adaptation (Stirman et al., 2019) from the FAIR titration that typically occurs over 8–9 months. The planned PRE-FAIR trial will explicitly examine whether parents’ needs align with this titration schedule.

#### Prioritizing Ancillary Needs Treatment Component

Some parents reported that reduced stress due to meeting ancillary needs also reduced their desire to use substances. Meeting parents’ ancillary needs is thus a key potential mechanism for preventing substance use and might be emphasized earlier in PRE-FAIR compared to FAIR. Of note, tradeoffs or variation in how clinicians and parents spend their time focusing on each FAIR domain (substance use, parenting, mental health, and ancillary needs) will be modeled in a future simulation study and tested in the PRE-FAIR trial.

Practically, as PRE-FAIR clinicians expand services to new counties and new system partners, careful attention should be paid to the time and skills clinicians need for generating relationships with community partners that can help meet parents’ ancillary and other treatment component needs, such as mental health providers that can provide mental health support to parents after they graduate PRE-FAIR. Clinicians often reflected on how they relied upon their network of community partners and fellow clinicians to identify resources, which helped mitigate the balancing loop impact of low service availability on meeting parents’ ancillary needs (Figure 2). The PRE-FAIR partnership with Self-Sufficiency will provide an excellent opportunity to leverage existing resources. For example, a clinician reported leveraging their relationship with community partners to navigate waitlists: “Sometimes I’ve talked to community partners and I’ve said, ‘Can you guys start another class? Can there be another night that you guys do respite care?’ Because a lot of my parents need that.” Another clinician succinctly described their ingenuity and perseverance in establishing relationships: “We work closely with DHS to see what community partners DHS has, especially in rural communities. We call around. We learn from those other community members too what else is – because I get a lot of my information from other people.” However, as clinicians spend more time fostering community partnerships, they will have less time to engage parents (Supplementary Figure 2). The tradeoff between demands to clinicians’ time also will be examined in the simulation study and PRE-FAIR trial.

### Limitations

This study should be interpreted within the context of its limitations. First, the parent sample size might have been insufficient to extract all potential considerations for FAIR adaptation to PRE-FAIR, and insights might be specific to the current study sample. For example, interviews initially revealed few balancing loops, requiring consultation with the FAIR developer to clarify dynamics such as how parent mental health symptoms related to positive parenting practices (e.g., appropriate developmental expectations) and parenting deficits (e.g., limited parenting skills, neglectful parenting) (Figure 1E). Additional parent and clinician interviews could increase robustness of the CLD. However, there was consistency in the variables that operationalized FAIR engagement and the stories that operationalized the interconnectedness of these intervention strategies (i.e., feedback loops).

Second, the recruitment of only graduated FAIR parents might have led to bias in parent reports and limitations in the scope of insights. Parents shared overwhelmingly positive comments about FAIR when probed for critiques and suggestions, limiting insights on what might be improved for parents who graduated FAIR and what engagement strategies helped or hindered engagement for parents who discontinued. Of the suggestions that were offered, responses were not negative, but rather, for example, a request for more frequent and intensive training in life skills. Other suggestions tended to focus on factors outside of the clinician’s control, such as more funding for federal programs that support parents (e.g., SNAP/EBT). A particularly relevant suggestion for PRE-FAIR was to have FAIR provide additional services that might only be available to parents with an open DHS case, such as extra parenting classes or child care. Similar to previous trials of FAIR, which have extensively probed FAIR’s acceptability to parents, the PRE-FAIR trial will carefully examine whether PRE-FAIR is meeting parents’ needs or could be improved. Including only a sample of successfully graduated FAIR parents also might have limited insights around potentially necessary prevention adaptations. PRE-FAIR parents might have unique treatment goals, existing supports, or desired supports compared to the parents interviewed in the current study. Thus, the PRE-FAIR trial will be designed to capture these differences.

Relatedly, several interviewed parents did not graduate FAIR during their first enrollment in FAIR. They reflected on the more intensive treatments that were required for them prior to returning to FAIR. Because of the current study’s focus on making adaptations for a prevention model for parents with less severe baseline substance use, this theme was not highlighted in the current set of CLDs. The converse might be true in the PRE-FAIR parent population; parents might not quickly see the need for PRE-FAIR. As identified in this study, clinicians can apply flexible, creative engagement strategies to understand what aspects of PRE-FAIR parents might be most effective and best meet their needs. Alternatively, clinicians can refer parents to FAIR for a more intensive treatment. To address these potential limitations, attention will be paid to identifying additional feedback loops and engagement strategies during the PRE-FAIR trial.

### Future Research

Immediate next steps include understanding whether: (1) engagement strategies with PRE-FAIR parents differ from successful strategies with FAIR parents; (2) PRE-FAIR is acceptable to parents and results in positive parent and child outcomes, such as reductions in the initiation or escalation of parental opioid and/or methamphetamine use, or DHS outcomes such as removal of children from the home or new reports; and (3) PRE-FAIR implementation costs are sustainable. Adaptations made during PRE-FAIR implementation that were not identified in the current study will be recorded in order to inform future adaptation planning methods.

As noted above, a system dynamics simulation model also will be pursued. This model will support PRE-FAIR clinics in anticipating how PRE-FAIR dynamics, such as more frequent caseload turnover due to a shorter treatment duration, might affect clinical dynamics, such as how quickly new clinicians reach competency in PRE-FAIR clinical strategies and, consequently, how much time clinicians spend with parents. The simulation will be used to learn about potential tradeoffs in how clinicians spend their time, and how these tradeoffs might impact caseload size and claims reimbursement. Insights could thus guide training activities for new PRE-FAIR clinicians and clinic administrators.

Broadly, future research should explore the use of similar systems science-based approaches for planning intervention adaptation and implementation planning efforts. Studies should examine whether such strategies sufficiently identify requisite EBP component and implementation adaptations, and whether EBPs adapted with systems science strategies lead to improved population health outcomes as expected.

## Conclusion

Given the deleterious effects plaguing the child welfare system and families caused by the opioid and methamphetamine epidemics, there is an urgent need to develop preventive interventions that can address the myriad needs of parents at risk for substance abuse. Drawing on the limited EBPs available to address the treatment of this problem once the symptoms are severe, effective preventive interventions might be possible. Rigorous adaptation of EBPs can support efficacy of the interventions in new settings (e.g., community and school), geographic regions, and populations (e.g., prevention). Previous studies have noted the importance of carefully planning adaptation to reduce the likelihood of reduced efficacy or acceptability of the intervention by participants (Baumann et al., 2017; Rabin et al., 2018; Stirman et al., 2019). This study presented an innovative application of systems science methods to rigorously identify treatment components that should be maintained or modified, as well as implementation processes that might be affected by prevention adaptations. Insights from the current study will help investigators anticipate what EBP components might be adapted to better support prevention intervention efforts, while also anticipating which treatment components need to be carefully monitored and adapted at subsequent stages of prevention intervention implementation. Future research will evaluate the impact of prevention adaptations on key parent outcomes. Although parental opioid and/or methamphetamine use are leading public health concerns, effective preventive interventions, and the engagement of parents in these interventions, is possible. Future policy must support these efforts for a public health impact to be realized.

## Data Availability Statement

De-identified qualitative data is not readily available but might be made available upon request to be analyzed in collaboration with members of the investigative team. Causal Loop Diagram data will be made available by the authors. Requests to access the datasets should be directed to LS, lisas@oslc.org.

## Ethics Statement

The studies involving human participants were reviewed and approved by Oregon Social Learning Center Institutional Review Board. The patients/participants provided their written informed consent to participate in this study.

## Author Contributions

GC conducted all interviews, led all analyses, and drafted the initial manuscript. SC co-coded qualitative analyses, validated causal loop diagrams, and provided substantive edits to the manuscript. LS obtained funding, provided FAIR materials, and served as a validation source for manualized FAIR treatment components, and provided substantive edits to the manuscript. All authors agreed to be accountable for the content of the work.

## Conflict of Interest

LS is the developer of FAIR. She was not involved in any primary analyses or data management. The remaining authors declare that the research was conducted in the absence of any commercial or financial relationships that could be construed as a potential conflict of interest.

## Publisher’s Note

All claims expressed in this article are solely those of the authors and do not necessarily represent those of their affiliated organizations, or those of the publisher, the editors and the reviewers. Any product that may be evaluated in this article, or claim that may be made by its manufacturer, is not guaranteed or endorsed by the publisher.
