# Supplementary material for: Prevention Adaptation of an Evidence-Based Treatment for Parents Involved With Child Welfare Who Use Substances
Source: Front Psychol. 2021 Nov 19;12:689432. doi: 10.3389/fpsyg.2021.689432 (PMC8639490; doi:10.3389/fpsyg.2021.689432)
Supplement: Supplementary file 1 [file Data_Sheet_1.docx]

*Supplementary Material*

# Introduction to Supplementary Material

The CLDs derived from FAIR key informant interviews highlighted the integral nature of FAIR treatment strategies across all four FAIR key treatment components. Parent success in each treatment domain was perceived to facilitate short and long-term improvement in other domains (Supplementary Section 2). This finding is consistent with the FAIR logic model, and highlights the need to maintain FAIR’s four treatment domains in PRE-FAIR (substance use, parenting skills, mental health, ancillary needs; Supplementary Section 3).

The following feedback loops demonstrate dynamics essential to understanding how FAIR supports parents in making positive choices, including how FAIR’s core treatment domains interact dynamically over time (Supplementary Section 2). Essential clinical processes that are hypothesized to affect the treatment duration and clinical caseload capacity, such as clinician’s experience level and available time to work with parents, also are presented (Supplementary Section 4). The balancing and feedback loops related to clinician experience and time, in particular, are preliminary insights from the current study and will be tested further for robustness in the PRE-FAIR Hybrid I effectiveness-implementation trial (Curran et al. 2012).

# Concurrent (Endogenous) Parent Success Across FAIR’s Four Treatment Components

Engagement strategies directly and indirectly facilitated parent success across FAIR’s four key treatment components (substance use, parenting skills, mental health, ancillary needs). Five key reinforcing loops characterized how parents reached and maintained positive outcomes (Supplementary Figure 1 Panels A, B, C, D). The relationship between engagement and ancillary needs was described in the context of strengths-based engagement (Figure 1 Panel A; Manuscript Section 3.2.5). Two feedback loops in particular highlighted the interdependency of positive parent outcomes across the four primary treatment components and are described below (Supplementary Figure 1 Panels B, C).

## Mental Health and Sobriety (Supplementary Figure 1 Panel B)

As parents felt more empowered and “worthy” of meeting their treatment goals, they felt capable of making additional positive choices, which improved their mental health. Through this sense of empowerment, combined with the sobriety skill building obtained through high-quality treatment, parents increasingly made positive choices about their health, leading to the increased likelihood that they achieved and maintained sobriety. As parents achieved and maintained sobriety, they recognized their success and felt further empowered to continue making positive choices. Empowerment was especially important for parents who had recently achieved sobriety: “Just him congratulating me as well every step of the way was very helpful for me because again, I didn’t have those feelings within myself. Because we always want to be further ahead than we actually, or where we think our life should look like this, but really it’s like this and just being reassured through the steps of my early recovery.”

## Mental Health and Parenting Practices (Supplementary Figure 1 Panel C)

FAIR teaches and supports parents to use positive parenting practices. As parents saw that they had the skills to employ positive parenting practices, their mental health increased. As parents experienced less stress related to challenging child behaviors, they gained a greater sense of confidence in their capacity to be positive parents: “Mainly, the services I utilized with [clinician] was parenting stuff. We went over a lot of that and mainly just support through just staying clean and knowing that I could do that. I had a lot of self-doubt, so he helped me with feeling more confident and stuff about that.” Clinicians were keenly aware of this feedback loop:

I’ll point out to how it translates back to just other mental health solutions, other parenting solutions. Oftentimes getting these things up and running can feel like work… So, reframing that for them is like: “Yeah, or we can look at this as an opportunity to be creative, or we can look at this as an opportunity to see your self-care as a priority, taking care of yourself as a priority so that you can be an epic mom or be an epic dad.”

## Strong Relationships and Sobriety (Supplementary Figure 1 Panel D)

Both clinicians and parents reported that strong, reliable, and positive social (e.g., friends, family members) and professional (e.g., community mental health clinician, physician) relationships were imperative to parents reaching their treatment goals in each domain. Supplementary Figure 1 (Panel D) and Figure 2 show the integral nature of strong relationships within virtuous feedback loops in which parents achieved and maintained sobriety, improved their mental health, and improved parenting practices. Strong relationships also directly increased the likelihood that parents increasingly acquired ancillary supports, which often provided further opportunities to foster strong peer relationships in long-term community settings (e.g., substance use recovery peer support groups, religious settings). As parents achieved and maintained sobriety, they were more equipped to re-build relationships or acquire relationships that could help them maintain sobriety. Clinicians reported helping parents attune to the need to strengthen relationships, but also followed each parent’s lead about which relationships were priorities to rebuild:

Of course, it’s up to the parent, but the parent sometimes—especially if it’s their first time being engaged, they don’t even know what’s going to be a good fit for them. All the time that we work with our parents from day one, we’re trying to build those supports. That’s what I do. I try to build those supports almost immediately. We ask the question, who is your support?

Parents also reported looking forward to re-building relationships, including relationships with their children, as they achieved sobriety:

We went over a lot of stuff with my daughter because she was old enough to understand the stuff that I put her through, and she was living with her grandma nearly coming back into my home so there was a lot of acting out and stuff on her side…She had some anger and stuff like that towards me and towards her grandma… We went over a lot of that kind of stuff, and just how to deal with that in a productive way, and to make our bond and our relationship a little stronger moving forward.

Graduated FAIR parents recognized the role of relationships in maintaining their sobriety as well. Reflecting on the value of a community-based group for people in recovery, one parent recalled how important those peer relationships were for them to achieve and maintain sobriety: “What I’ve realized is that I can’t do it alone. I need other people in my life that understand where I am today.”

Similarly, strong relationships helped parents improve their mental health through reducing stress and improving their sense of confidence. In turn, parents felt empowered to foster and maintain strong relationships.

# Maintaining the Four FAIR Treatment Domains

First, the utility of FAIR’s four treatment domains was validated by perceived interconnections between each component and outcomes such as sobriety and reduced parenting deficits. Successful parent outcomes in one domain were perceived to perpetuate a virtuous (i.e., reinforcing) feedback loop with the other three domains. The reinforcing nature of the feedback loops suggested that improvement in one domain not only leads to short-term improvements in other domains, but that improvement in each domain can beget long-term maintenance of parents’ overall health and well-being. For example, parents could improve their parenting practices, but without sufficient ancillary supports to reduce stress related to basic needs such as shelter and housing, their mental health is unlikely to improve enough to fully implement their improved parenting practices. These insights are consistent with recent studies based on national data that highlight the importance of parental mental health and social supports in preventing child neglect (Kepple and Parker 2021). The synergy across treatment domains identified in the current study is consistent with research recognizing the potential synergy between parental substance use and parenting practices (Bosk et al. 2019; Dawe et al. 2003). Results suggest that PRE-FAIR clinicians should maintain fidelity to the FAIR model, synergistically delivering all four domains to help parents avoid initiating vicious cycles that might lead to fewer positive choices about their health and sobriety (e.g., substance use, Supplementary Figure 1 Panel B).

The diversity of FAIR’s treatment components helps break the undesirable feedback loop of substance use behaviors. The conceptual model described by the CLDs suggested that PRE-FAIR treatment strategies can provide parents with the supports that prevent initiation of this undesirable substance use feedback loop. However, the ways in which these evidence-based treatment strategies are delivered and the order of delivery for each strategy might vary in PRE-FAIR compared to FAIR. As one clinician surmised, “The principles are pretty consistent and stay the same, just how we talk about it and how we shape that language around it is really what changes.” Similarly, while the types of relationships that PRE-FAIR parents might desire to build or re-build might differ from FAIR parents, clinicians identified relationships to be a crucial factor for substance use prevention: “Prevention is just also getting supports in there. That’s really, really important at the very beginning like, ‘Who are your supports there, and are they appropriate?’” In short, the inherent flexibility of each treatment activity to tailor delivery for each parent can be carried forward to PRE-FAIR.

# Behaviors Over Time to be Explored in Future Simulations

Multiple behaviors over time that might facilitate PRE-FAIR’s effectiveness will be explored in future research, including: parent’s time in treatment; discontinuation and graduation rates; treatment dosage and duration; parental substance use. For example, insights derived from FAIR clinician and administrator interviews suggested that parents’ treatment timelines will potentially affect PRE-FAIR billing (e.g., fewer services billed per parent over the course of treatment) and caseloads (e.g., more frequent caseload turnover and thus potentially shorter waitlists). A quantitative system dynamics model is being developed to understand the clinical and administrative impacts of a potentially shorter and less intensive treatment duration on clinical factors such as average caseload and clinician burnout, and administrative factors such as workforce size and clinic costs. Supplementary Figure 2 demonstrates some of the clinical workforce processes that will be simulated.

Supplementary Material References

Bosk, Emily A., Ruth Paris, Karen E. Hanson, Debra Ruisard, and Nancy E. Suchman. 2019. “Innovations in Child Welfare Interventions for Caregivers with Substance Use Disorders and Their Children.” *Children and Youth Services Review*, 101: 99-112. https://doi.org/10.1016/J.CHILDYOUTH.2019.03.040.

Curran, G M, M Bauer, B Mittman, J M Pyne, and C Stetler. 2012. “Effectiveness-Implementation Hybrid Designs: Combining Elements of Clinical Effectiveness and Implementation Research to Enhance Public Health Impact.” *Medical Care* 50 (3): 217–26. https://doi.org/10.1097/MLR.0b013e3182408812.

Dawe, S, P H Harnett, V Rendalls, and P Staiger. 2003. “Improving Family Functioning and Child Outcome in Methadone Maintained Families: The Parents Under Pressure Programme.” *Drug and Alcohol Review* 22 (3): 299–307. https://doi.org/10.1080/0959523031000154445.

Kepple, Nancy J, and Amittia Parker. 2021. “Examining Unique Substance-Related Risk Profiles for Neglectful Behaviors among Parents with and without Clinical Depression.” *Children and Youth Services Review*, 125: 105987. https://doi.org/https://doi.org/10.1016/j.childyouth.2021.105987.

**Supplementary Figure 1: Feedback Loops Describing FAIR Logic Model**

| 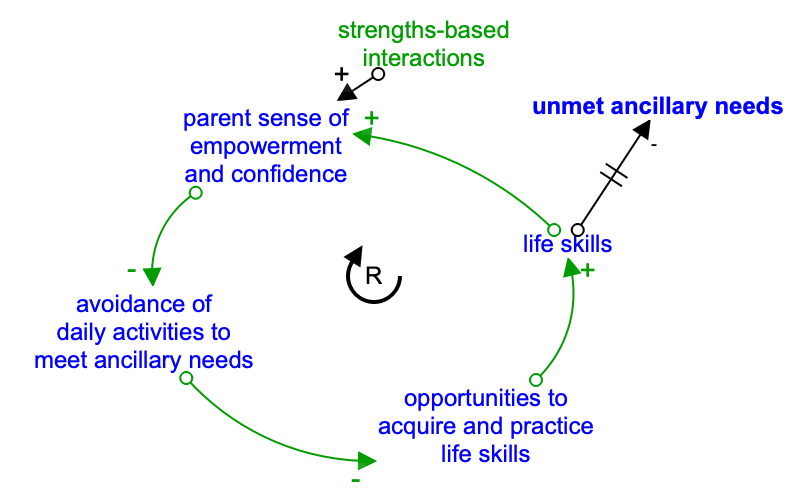   1. Reinforcing Loop: Strengths-based interactions build parent empowerment and life skills | 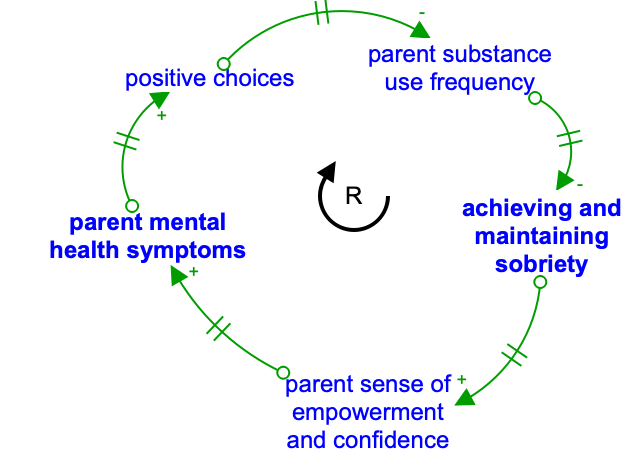   1. Reinforcing Loop: Endogenous parent outcomes: Parent mental health and sobriety |
| --- | --- |
| 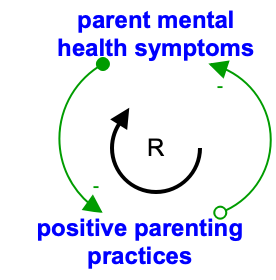   1. Reinforcing Loop: Endogenous parent outcomes: Parent mental health and parenting practices | 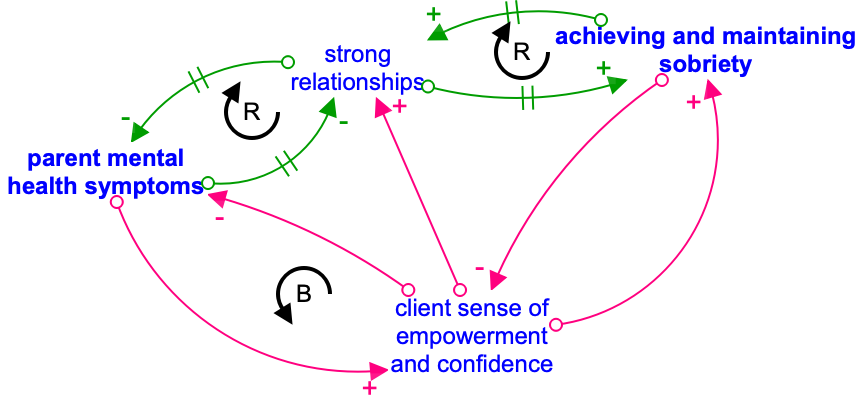   1. Reinforcing Loops: Strong relationships beget improvement across treatment domains   Balancing Loop: Mental health and sobriety |
| 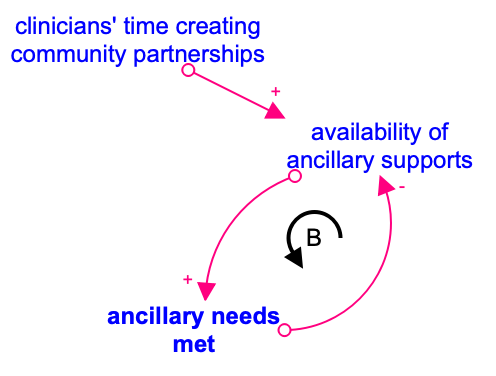   1. Balancing Loop: Available ancillary supports |  |
| Note: Engagement strategies designated in green. Outcomes related to the four primary FAIR treatment domains designated in bold. Reinforcing loops indicated in green with R clockwise arrow, and Balancing loops indicated in pink with B counter-clockwise arrow. Arrows with a + sign indicate that the variables either both increase or both decrease when there is a change. Arrows with a – sign indicate that as one variable increases, the other decreases, or vice versa. | |

**Supplementary Figure 2: Example Causal Loop Diagram to Inform Simulation of Potential PRE-FAIR Implications for Clinicians and Clinic Administrators**


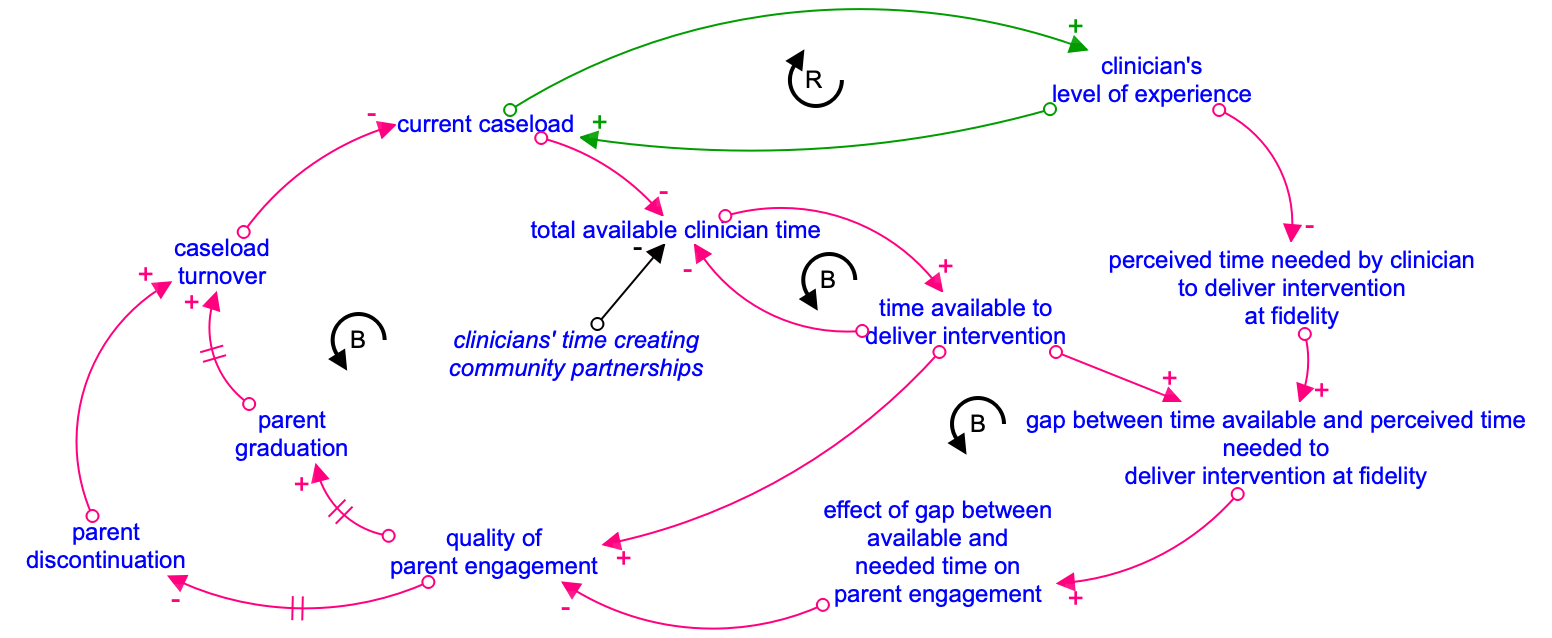


Note: Reinforcing loops indicated in green with R clockwise arrow, and Balancing loops indicated in pink with B counter-clockwise arrow. Arrows with a + sign indicate that the variables either both increase or both decrease when there is a change. Arrows with a – sign indicate that as one variable increases, the other decreases, or vice versa.
